# Metabolic Acidosis in Chronic Alcoholism: When It’s Not Just Diabetic Ketoacidosis

**DOI:** 10.7759/cureus.96632

**Published:** 2025-11-11

**Authors:** Robert Li, Jennifer Han, Harpreet Sidhu

**Affiliations:** 1 Endocrinology, Diabetes and Metabolism, University of California Los Angeles David Geffen School of Medicine, Los Angeles, USA; 2 Nephrology, University of California Los Angeles David Geffen School of Medicine, Los Angeles, USA

**Keywords:** alcoholic ketoacidosis, diabetic ketoacidosis (dka), diagnostic error, ketacidosis, metabolic ketoacidosis

## Abstract

Metabolic acidosis with ketosis is a medical emergency. Due to divergent management strategies, prompt differentiation is required between diabetic ketoacidosis (DKA), alcoholic ketoacidosis (AKA), and euglycemic diabetic ketoacidosis (euDKA). We present the case of a 28-year-old woman with heavy alcohol use who presented with severe metabolic acidosis, ketosis, and hyperglycemia who was initially treated for DKA. Unexpected lab discrepancies in the course of treatment including hypoglycemia despite dextrose infusion raised suspicion for AKA. Elevated C-peptide, normal HbA1c, and absent anti-islet antibodies confirmed the diagnosis. This case underscores the importance of recognizing AKA’s distinct pathophysiology and avoiding inappropriate insulin therapy. A systematic approach to metabolic acidosis with ketosis, emphasizing clinical context and lab correlation, is critical to prevent mismanagement.

## Introduction

Metabolic acidosis with ketosis is a medical emergency that requires prompt recognition and initiation of appropriate management. Although diabetic ketoacidosis (DKA) is the most common presentation of metabolic acidosis, alcoholic ketoacidosis (AKA) and sodium glucose transporter-2 (SGLT-2) inhibitor-induced euglycemic DKA (euDKA) must also be considered [1,2]. Despite commonly presenting with ketosis and anion gap metabolic acidosis, DKA, AKA, and euDKA are fundamentally different in their pathophysiology, risk factors, and treatment [3,4]. Misdiagnosis can lead to inappropriate therapy, such as insulin-induced hypoglycemia in AKA or delayed SGLT-2 discontinuation in euDKA.

Classic DKA is well known to be the result of absolute insulin deficiency leading to uncontrolled lipolysis, hyperglycemia, and severe acidosis [5]. SGLT-2 inhibitor-induced euDKA, in contrast, arises from medication-induced glucosuria and relative insulin deficiency, even in non-diabetic patients using these medications for heart failure or chronic kidney disease [6,7]. Alcoholic ketoacidosis, on the other hand, typically develops following a binge episode in malnourished chronic alcohol users, where alcohol metabolism and starvation stimulate ketogenesis despite normal insulin levels [8]. The overlapping clinical presentations of these conditions combined with their divergent management strategies emphasizes the need for a systematic diagnostic approach.

## Case presentation

A 28-year-old previously healthy woman with no significant past medical history and on no prescription medications presented to the emergency department with three days of worsening non-bilious, non-bloody emesis. In the 24 hours prior to presentation, emesis frequency increased to hourly. Vital signs on presentation were notable for tachycardia (heart rate 138 beats per minute), tachypnea (respiratory rate 24 breaths per minute) and hypertension (blood pressure 175/95 mmHg). She was afebrile (temperature 36.7°C) with normal oxygenation of 97% on room air. Prior to the onset of symptoms, she had a 10-day history of heavy alcohol consumption with intake of 10-15 shots of liquor daily. Initial laboratory evaluation revealed multiple metabolic derangements including acidemia (Table 1), high anion gap metabolic acidosis (Table 2), and ketonuria (Table 3). 

**Table 1:** Venous Blood Gas Showing Severe Acidemia (LL): Data is critically low (H): Data is abnormally high

Lab	Patient Value	Reference Range
pH, Venous	7.11 (LL)	7.32 - 7.42
PCO2, Venous	20 (LL)	40 - 50 mmHg
PO2, Venous	104 (H)	25 - 40 mmHg
HCO3, Venous, Point of Care	6.4 (LL)	24.0 - 28.0 mmol/L

**Table 2:** Serum Chemistry Showing an Anion Gap Metabolic Acidosis (LL): Data is critically low (L): Data is abnormally low

Lab	Patient Value	Reference Range
Na	134	134 - 146 mmol/L
K	4.0	4.0 mmol/L
Cl	90	100 - 110 mmol/L
CO2	<10 (LL)	20 - 31 mmol/L
Anion Gap	Unable to Calculate	5 - 14 mmol/L
Glucose	201 (H)	74 - 106 mg/dL
Phosphorus	<1.0 (LL)	2.5 - 4.5 mg/dL
Lipase	90 (H)	12 - 53 U/L
Lactate	2.6 (H)	0.3 - 2.0 mmol/L
Beta Hydroxybutyrate	13.90 (H)	0.02 - 0.27 mmol/L

**Table 3:** Urinalysis Showing Severe Ketonuria !: Data is abnormal

Lab	Patient Value	Reference Range
Glucose, Urinalysis	Negative	Negative
Ketones, Urinalysis	>150 mg/dL !	Negative, Trace

Evaluation for the underlying causes of a high anion gap metabolic acidosis showed elevated serum ketones, elevated serum lactate, and hyperglycemia (Table 2). Uremia was not present (Table 2). Ethanol, methanol, and salicylate levels were not checked.

Due to the presence of a high anion gap metabolic acidosis with hyperglycemia, elevated serum ketones, and ketonuria, the patient was diagnosed with diabetic ketoacidosis and admitted for treatment. Intravenous insulin, intravenous fluids, and electrolyte replacement were started with resolution of the anion gap metabolic acidosis after six hours of treatment (Table 4). The patient was kept nothing per mouth (NPO) until Day 2 at 18:05 (Table 4).

**Table 4:** Timeline of Laboratory Changes After Starting Treatment (LL): Data is critically low (L): Data is abnormally low (H): Data is abnormally high (HH): Data is critically high

Time	Day 1, 23:54	Day 2, 04:21	Day 2, 06:23	Day 2, 14:04	Day 2, 18:05
Sodium (134 - 146 mmol/L)	134	125 (L)	130 (L)	137	140
Potassium (3.5 - 5.2 mmol/L)	4.0	2.9 (L)	3.5	3.0 (L)	3.6
Chloride (100 - 110 mmol/L)	90 (L)	96 (L)	98 (L)	104	105
Carbon Dioxide (20 - 31 mmol/L)	<10 (LL)	<10 (LL)	16 (L)	23	22
Anion Gap (5 - 14 mmol/L)	-	-	16 (H)	10	13
Glucose, Serum (74 - 106 mg/dL)	201 (H)	948 (HH)	555 (HH)	187 (H)	68 (L)

During the course of treatment, several unexpected findings emerged, including marked glucose discrepancies between serum glucose and capillary glucose levels (Table 5) and development of hypoglycemia despite dextrose infusion (Table 6). Further evaluation revealed a normal C-peptide and A1c (Table 7).

**Table 5:** Unexpectedly Elevated Serum Glucose During Treatment (HH): Data is critically high (H): Data is abnormally high

Time	Day 1, 23:54	Day 2, 02:48	Day 2, 03:37	Day 2, 04:21	Day 2, 04:32	Day 2, 05:23	Day 2, 06:23	Day 2, 06:32
Glucose, Serum (mg/dL)	201 (H)	-	-	948 (HH)	-	-	555 (HH)	-
Glucose, Point of Care (mg/dL)	-	161 (H)	190 (H)	-	181 (H)	172 (H)	-	196 (H)

**Table 6:** Development of Hypoglycemia During Treatment (L): Data is abnormally low

Time	Day 2, 16:04	Day 2, 17:17	Day 2, 18:05	Day 2, 18:35	Day 2, 20:14
Glucose (mg/dL)	-	-	68 (L)	-	-
Glucose, Point of Care (mg/dL)	80	98	-	118	92
Dextrose 5% in water (D5W) rate (ml/hr)	250	250	250	250	250
Insulin infusion rate (units/hr)	1.5	0.3	0.3	0.3	0

**Table 7:** Chemistry Showing Normal Insulin Production (C-peptide) and Long-Term Euglycemia (Hemoglobin A1c)

Lab	Patient Value	Reference
C-peptide	3.7	0.81 - 3.82 ng/mL
Hemoglobin A1c	5.10%	4.1 - 6.5%

Upon further investigation, it was found that all prior venous blood draws were done on the same line as the dextrose 5% in water (D5W) infusion, thus pointing to an iatrogenic cause for the hyperglycemia. In light of this fact, her presentation of anion gap metabolic acidosis, ketonuria, elevated serum ketones, significant alcohol intake, and euglycemia pointed to a diagnosis of AKA rather than DKA. Insulin infusion was immediately discontinued and subcutaneous insulin was not started. The patient was started on a general diet and maintained euglycemia throughout the remainder of hospitalization with complete normalization of all electrolyte abnormalities. Subsequent testing showed negative diabetes autoantibodies, ruling out type 1 diabetes.

## Discussion

This patient was incorrectly diagnosed and treated due to a misdiagnosis of diabetic ketoacidosis. Inappropriately drawn labs showing hyperglycemia when none was actually present mistakenly directed treatment towards use of intravenous insulin, leading to hypoglycemia. It is important to review the causes, presentation, and treatment of high-anion-gap metabolic acidosis with ketosis. 

Differentiating the types of ketoacidoses

The evaluation of high anion gap metabolic acidosis with ketosis requires careful differentiation between DKA, AKA and euDKA, as management differs substantially. Whereas DKA results from absolute insulin deficiency, most commonly in the setting of type 1 diabetes [1], AKA typically occurs in chronic alcohol users after a binge period followed by vomiting and starvation [3]. Euglycemic DKA, in contrast, develops from the effects of SGLT-2 inhibitors, which promote urinary glucose excretion and can create a state of relative insulin deficiency and increased lipolysis [4] in both diabetics and non-diabetics [7].

In this case, the patient had positive history for chronic alcohol binge use but no history of diabetes or use of SGLT-2 inhibitors. Confounding the presentation was the presence of hyperglycemia which raised suspicion of diabetes and diabetic ketoacidosis rather than alcoholic ketoacidosis.

Laboratory differences

While DKA, AKA, and euDKA all present with ketonemia and high anion gap metabolic acidosis, key laboratory differences help distinguish between the conditions. Glucose levels are typically markedly elevated (>250 mg/dl) in DKA compared to normal or mildly elevated (100 - 200 mg/dl) in AKA and normal in euDKA [2,5]. C-peptide levels are typically low or zero in DKA whereas they are normal (0.8 - 3.1 ng/ml) in AKA [9] and low to normal in euDKA (0.4-1.0 ng/mL) [10]. The presence or absence of glucosuria can distinguish between AKA (no glucosuria unless hyperglycemia occurs), euDKA (marked glucosuria), and DKA (present) [9]. 

In this case, iatrogenic hyperglycemia led to a mistaken diagnosis of diabetic ketoacidosis despite the absence of glucosouria (Table 3). A more careful analysis of the laboratory testing should have raised suspicion that diabetic ketoacidosis was not the correct diagnosis. 

Management strategies

Due to the fundamentally different pathophysiologies, treatment for DKA, AKA, and euDKA differs widely. Treatment of DKA is with insulin therapy, intravenous fluids, and aggressive replacement of electrolytes [5]. AKA management primarily focuses on fluid resuscitation with dextrose-containing fluids to suppress ketogenesis and thiamine repletion with specific avoidance of insulin due to the risk of inducing hypoglycemia [8]. Management of euglycemic DKA involves discontinuing the SGLT-2 inhibitor and use of low-dose insulin infusion with careful glucose monitoring [1].

In this patient, intravenous fluids with dextrose were started as part of the DKA treatment protocol. As intravenous fluids are also the treatment for AKA, the patient had a quick resolution of her anion gap metabolic acidosis. However, the second component of DKA treatment, intravenous insulin, led to hypoglycemia which would have caused significant morbidity had it not been recognized. 

A summary of the different types of ketoacidoses, their presenting labs, and treatments is listed in the table below (Table 8).

**Table 8:** Diagnosis and Management of Ketotic Metabolic Acidosis NADH: Nicotinamide adenine dinucleotide BHB: Beta hydroxybutyrate SGLT-2: sodium/glucose cotransporter 2 T1DM: type 1 diabetes

Feature	Alcoholic Ketoacidosis (AKA)	Classic Diabetic Ketoacidosis (DKA)	SGLT-2-Induced Euglycemic DKA (Non-Diabetic)
Primary Cause	Chronic alcohol use + starvation	Absolute insulin deficiency (T1DM/T2DM)	SGLT-2 inhibitor use (e.g., empagliflozin)
Pathophysiology	NADH excess → BHB-dominant ketosis	Insulin deficiency → lipolysis → ketosis + hyperglycemia	Glucosuria → "starvation-like" ketosis
Blood Glucose	Normal/low (70–200 mg/dL)	High (>250 mg/dL)	Normal/mildly high (70–250 mg/dL)
Anion Gap	Elevated	Elevated	Elevated
pH/HCO₃⁻	pH <7.3, HCO₃⁻ <10	pH <7.2, HCO₃⁻ <10	pH <7.3, HCO₃⁻ <18
Ketones	↑↑ BHB (>3 mmol/L)	↑↑ BHB + acetoacetate	↑↑ BHB (>3 mmol/L)
C-Peptide	Normal/high	Low/absent	Low/normal (relative insulin deficiency)
A1c	Normal (<5.7%)	Elevated (≥6.5%)	Normal (<5.7%)
Urine Glucose	Negative (unless hyperglycemic)	Positive	Positive (SGLT-2 effect)
Key Risk Factors	Alcohol binge + vomiting	T1DM, missed insulin doses	SGLT-2 use (even without diabetes)
Treatment	D5W + thiamine (NO insulin)	Insulin + fluids	Stop SGLT-2 + low-dose insulin
Electrolytes	Severe K⁺/Mg²⁺/PO₄³⁻ depletion	K⁺ shifts (initially high, then low)	Mild K⁺/PO₄³⁻ depletion

## Conclusions

In this case, the patient presented with findings that were in retrospect consistent with AKA. However, due to errors in drawing blood leading to a mistaken diagnosis of hyperglycemia, insulin therapy was inappropriately administered and the patient developed hypoglycemia. The absence of glucosouria on presentation as well as the history of significant alcohol intake should have prompted a more critical evaluation of the correctness of the DKA diagnosis. This case highlights the importance of critically interpreting laboratory results in a clinical context, understanding the pathophysiology and management of the different types of ketoacidoses.
